# Effectiveness of the Internet-Based Versus Face-to-Face Interaction on Reduction of Tobacco Use Among Adults: A Meta-Analysis

**DOI:** 10.7759/cureus.19380

**Published:** 2021-11-08

**Authors:** Ravi Kant, Poonam Yadav, Mukesh Bairwa

**Affiliations:** 1 General Medicine, All India Institute of Medical Sciences, Rishikesh, Rishikesh, IND; 2 College of Nursing, All India Institute of Medical Sciences, Rishikesh, Rishikesh, IND; 3 Internal Medicine, All India Institute of Medical Sciences, Dehradun, Dehradun, IND

**Keywords:** tobacco, smoking cessation, meta-analysis, internet, adults

## Abstract

Literature reported the effectiveness of internet-based interventions over face-to-face interaction on tobacco quitting; however, limited sample size reinforces to integrate and analyze these studies' findings to reach a single conclusion. Therefore, we evaluated the effectiveness of the internet as an intervention approach versus face-to-face interaction on reducing tobacco use among adults. A systematic search was performed through various electronic databases such as Medline, PsychInfo, PubMed, Embase, Cochrane Central Register of Controlled Trials (CENTRAL), ResearchGate, Google Scholar, and Academia. Reference lists of the eligible articles were also screened. Full-text articles were included as per eligibility criteria (PICO framework). No ethnicity restriction was applied. A total of 13 studies were selected for meta-analysis, with 3852 and 3908 participants in intervention and control groups, respectively. Forest plot favours the intervention group at one month follow up for tobacco quitting (OR: 2.37, CI: 1.86-3.02, *P*=0.00001, I2=0%), at three months (OR: 1.88, CI: 1.48-2.40, *P*=0.00001, I2=42%) at six months (OR: 2.02, CI: 1.64-2.50, *P*=0.00001, I2=38%) and at one year of follow-up (OR: 1.43, CI: 1.18-1.74, *P*=0.00001, I2=36%) comparing to control group. Conclusively, internet and web-based interventions are highly useful in tobacco quitting at one month, three months, six months, and one year of follow-up compared to face-to-face interaction or no intervention, although the level of evidence was moderate. Additionally, limited trials in developing countries, arising need for research on internet use for tobacco control in developing countries.

## Introduction and background

Tobacco use is the leading cause of avertible and premature deaths worldwide. The burden of tobacco-related disease is increasing in developed and developing countries as well [1]. Interestingly, the deaths are declining in developed countries, and the burden is shifting to developing countries [2]. However, tobacco consumption pattern varies across gender; male vs. female, domicile; rural vs. urban, regions, cultural practices, and family income [3]. Men are more frequently (23%) indulging in tobacco use than their counterparts (3%) [4]. Quitting any form of smoking is challenging and involves physiological, psychological, and many other factors, including social and environmental milieu to become successful [5]. In the case of smoking cessation, the best use of positive and negative reinforcements helps alleviate the withdrawal symptoms, and the role of behavioral approaches in smoking cessation cannot be denied [6].

Over the years, many innovative forms of internet-based approaches have been tried to quit tobacco use globally. The use of health communication and internet-based interventions like tailored computerized programs, text messages, mobile or telephone, and WhatsApp for reminder or call, app-based intervention, chat-based instant messaging, video assistance using the website and mobile [5] and use of social media, has been vividly used in recent decades to quit smoking among different age groups [7]. Although there is ample research and data regarding the potential influence of the media [7], face to face health education, cognitive behavior therapy, motivational influences, and nurses-assisted counseling [4], on behavioral changes among smokers, there are scanty reports on the internet use or behavioral interventions. They are neither planned nor conducted rigorously to indicate firm evidence of any encouraging effects on health outcomes.

Interestingly, the internet and other electronic platforms are abundantly present in this era and have almost become part and parcel of the health care system [2]. A medical expert with just a computer device and internet access, and some necessary handling skills can reach many people and communicate inexpensively. Though the effectiveness of internet-based and face-to-face interventions on quitting smoking are very well reported in the literature, every study carries one or another limitation in methodology and limited sample size. Therefore, it is required to integrate and analyze these studies' findings to reach a single conclusion. This study was planned to assess the effectiveness of the internet versus face-to-face interactions on reducing tobacco use among adults.

## Review

Methods

Data Sources and Search Strategy

The electronic databases, such as Medline, PsychInfo, PubMed, Embase, Cochrane Central Register of Controlled Trials (CENTRAL), Google Scholar, ResearchGate, and Academia, were explored. Reference lists of the eligible articles were also screened. All relevant studies available on the topic were included irrespective of time duration. The systematic search was restricted to studies published in the English language. The keywords were "smoker or smokers OR smoking," "tobacco" OR cigarette OR nicotine OR smoking cessation OR "tobacco consumption OR cessation, OR abstain* OR quit* OR stop* OR computer OR computer-aided design, OR internet, OR computer, OR networks, OR media, OR cellular phone OR mobile, OR text OR message* OR SMS, OR web, OR electronic mail OR Chat, OR video recording.

Eligibility Criteria (PICO Framework) for Participants

Inclusion criteria were adults aged more than 18 years who use the internet or face-to-face interventions to reduce or quit tobacco use. No ethnicity restrictions were applied. Exclusion criteria were Cochrane studies that compare the internet to face-to-face interventions with other interventions. 

Intervention: Internet interventions such as Phone, mobile, WhatsApp, Facebook, Online network group, Online Support group, text messaging, other internet media.

Comparator: Face-to-face interventions or no intervention in the comparator group. Face-to-face interventions include counseling, cognitive behavior therapy, or health education forms with control or routine care.

Outcome: Post-intervention tobacco quitting - number of participants quitting tobacco after the intervention (internet use).

Study design: This study is based on randomized controlled trials.

Time frame: No restriction to the time frame was applied

Screening of Eligible Studies

**Figure 1:**
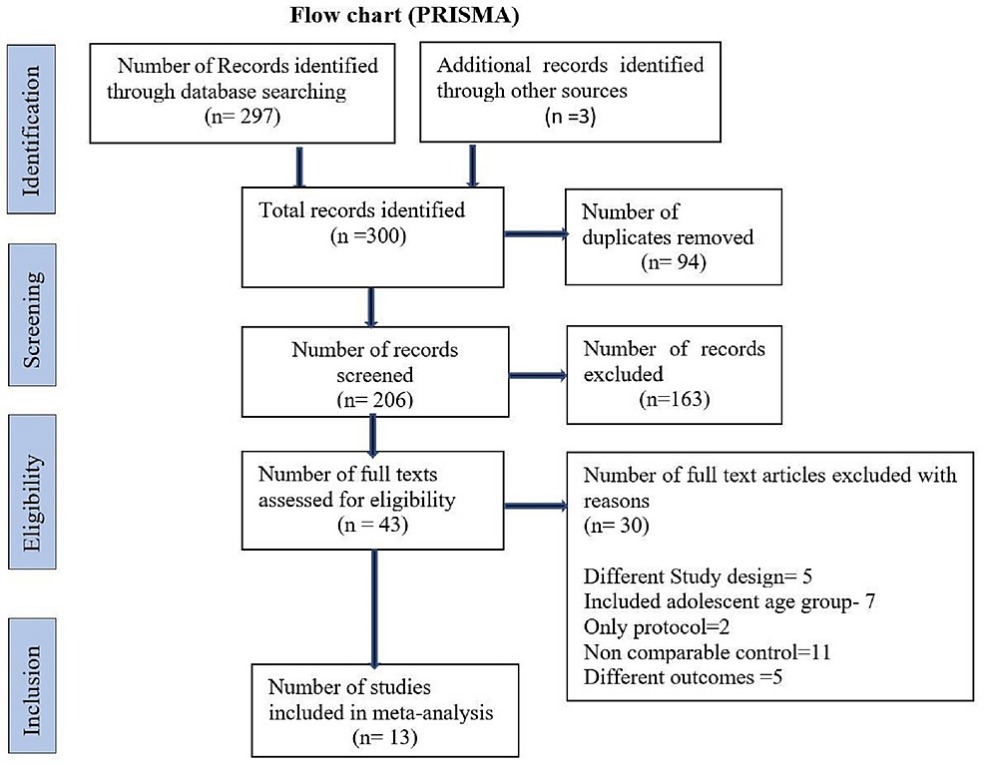
Flow chart (PRISMA) PRISMA: Preferred Reporting Items for Systematic Reviews and Meta-Analyses

A systematic search was done by two reviewers independently. After searching, studies were screened with titles and abstracts of respective studies. All selected studies were imported to Rayyan, a free web-based software. Two reviewers (PY and RK) screened the full text of articles based on eligibility criteria determined as per review protocol. Any relevant discrepancy has been resolved by consensus with the help of a third reviewer (MB). We adhered to the guidelines of Preferred Reporting Items for Systematic Review and Meta‑Analysis (PRISMA) 2009 [8]. PRISMA flow chart displays all the steps followed in the inclusion and exclusion of studies (Figure 1)

Eligible studies were exported to RevMan software 5.4 (The Cochrane Collaboration, London, UK) [9] for data analysis. Forest plots have been created to present the results with Odds ratio (OR), confidence interval (CI), and effect size.

The GRADE approach was also followed to explore the quality of evidence on high, moderate, and low levels [10]. RevMan files were exported to the GRADE Profiler to assess the quality of studies and create a "Summary of Findings" table (Table 1).

**Table 1:** Summary of findings table ^a^Wide confidence interval ^b^Heterogeneity OR: Odds ratio, CI: confidence interval, RCTs: randomized controlled trials

Outcomes	Anticipated absolute effects (95% CI)		Relative effect (95% CI)	No of participants (studies)	Certainty of the evidence (GRADE)
	Events in the control group	Events in the internet intervention group			
Tobacco quit at 1 month follow up	105 per 1,000	217 per 1,000 (179 to 261)	OR 2.37 (1.86 to 3.02)	2531 (5 RCTs)	⨁⨁⨁◯ MODERATE^a^
Tobacco quit at 3 months follow up	164 per 1,000	269 per 1,000 (225 to 320)	OR 1.88 (1.48 to 2.40)	1733 (5 RCTs)	⨁⨁⨁◯ MODERATE^b^
Tobacco quit at 6 months follow up	125 per 1,000	224 per 1,000 (190 to 263)	OR 2.02 (1.64 to 2.50)	2774 (6 RCTs)	⨁⨁⨁◯ MODERATE^a^
Tobacco quit at one year follow up	187 per 1,000	248 per 1,000 (214 to 286)	OR 1.43 (1.18 to 1.74)	2757 (6 RCTs)	⨁⨁⨁◯ MODERATE

Data Extraction

Two reviewers (PY and RK) extracted the data from the full text of eligible studies. Corresponding authors of included studies were contacted for the relevant data. Data excel sheet was prepared to note the characteristics of selected studies. It includes the author’s name with publication year, country, sample size, the mean age of participants, male to female ratio, baseline tobacco consumption, and follow-up period after the intervention (Table 2).

**Table 2:** Baseline characteristics of included studies

Author/year	Country	Interventions	Sample size intervention/control group	Mean age of participants (years)	Intervention/control group male/female (%)	Baseline cigarette consumption	Follow-up
Brendryen and Kraft [11]	Norway	A digital multi‐media intervention consists of more than 400 contacts by email, Web pages, interactive voice response, and short message service technology	200/200 smokers	Intervention - 35.9±10.0	Intervention - 49.2/50.8%	Intervention 18.3±5.9 cigs/day	1, 3, 6, and 12 months
				Control - 36.4±10.5	Control - 50.2/49.8 (%)	Control 18.1±5.8 cigs/day	
Brendryen et al. [12]	Norway	A digital multi‐media intervention consists of more than 400 contacts by email, Web pages, interactive voice response, and short message service technology without nicotine therapy	144/146 smokers	Intervention - 39.5±11	Intervention - 50/50 (%)	Intervention: 16.6±7.2 cigs/day	1, 3, 6, and 12 months
				Control - 39.7±10.8	Control - 50/50 (%)	Control 17.6±7 cigs/day	
Burford et al. [13]	Australia	A computer-generated photoaging intervention with no treatment group	80/80 smokers	Intervention - 24.2±4.1	Intervention - 31.3/68.7 (%)	Range- <1 to <21; intervention - 36.3%; smoked 11-20 cigs/day	SIx months
				Control - 25.1±4.1	Control - 43.8/56.2 (%)	Control - 33.8%; smoked 11-20 cigs/day	
Clark et al. [14]	United States	Internet resources for smoking cessation compared with written self-help material	85/86 smokers	Intervention - 57.8±5.2	Intervention - 54/46 (%)	Range - <10 to <31; intervention - 48%; smoked 11-20 cigs/day	One year
				Control - 57.0±5.3	Control - 48/52 (%)	Control - 44%; smoked 11-20 cigs/day	
Calhoun et al. [15]	United States	Internet intervention and telehealth medication clinic combined with a telehealth medication clinic for nicotine replacement therapy	205/203 smokers	Intervention - 43.3±13.6	Intervention - 85/15 (%)	Intervention - 15.7±8.8 cigs/smoking day	Three months and 12 months
				Control - 42.6±14.3	Control - 84/16 (%)	Control - 14.6±8.5 cigs/smoking day	
Elfeddali et al. [16]	Netherlands	Web-based computer-tailored programs	190/202	Intervention - 40.75±11.48	Intervention - 36.7/63.3 (%)	Intervention - 19.89±9.36) cigs/smoking day	Twelve months
				Control - 40.68±11.81	Control - 40.1/59.9 (%)	Control - 19.85±8.39 cigs/smoking day	
Japuntich et al. [17]	United States	The website which provided information on smoking cessation as well as support	140/144 smokers	Intervention - 40.6±12.4	Intervention - 45/55 (%)	Intervention - 21.1±9.5 cigs/smoking day	Six months
				Control - 41.0±11.8	Control - 45.1/54.9 (%)	Control - 22.1±10.2 cigs/smoking day	
Lawrence et al. [18]	United States	Personalized smoking cessation through an online life magazine	257/260 smokers	Intervention - 20.1±1.6	Intervention - 24.6/75.4 (%)	Intervention - 3.8±4.7) cigs/smoking day	30 weeks
				Control - 19.8±1.6	Control - 29.6/70.4 (%)	Control - 4.2±5.0	
McDonnell et al. [19]	Korea	Internet self-help smoking cessation program	272/315 smokers	Total 35 years (mean)	Total - 12/88 (%)	Total - 14 cigs/smoking day	Twelve months
Oenema et al. [20]	Netherlands	An internet-delivered computer-tailored lifestyle intervention	1080/1079 smokers	Intervention - 43.1±10.4	Intervention - 46/54 (%)	NA	One month
				Control - 44.1±10.4	Control - 47/53 (%)		
Pisinger et al. [21]	Denmark	Interactive, individual advice, newly developed by the Research Centre	476/442 smokers	Intervention - 49.63±16	Intervention - 36.8/63.2 (%)	Intervention - 18.12±10 cigs/smoking day	Twelve months
				Control - 46.97±17	Control - 36.6/63.4 (%)	Control - 16.25±8 cigs/smoking day	
Smit et al. [22]	Netherlands	A computer-tailored smoking cessation intervention through the Internet	552/571	Intervention - 48.4±12.2	Intervention - 45.8/54.2 (%)	Intervention - 20.8±13.7 cigs/smoking day	One month and six months
				Control - 48.8±12.3	Control - 49.4/50.6 (%)	Control - 20.4±11 cigs/smoking day	
Swartz et al. [23]	United States	A video-based internet site for smoking cessation and motivational materials	171/180 smokers	Intervention- control - 18-70 years (range)	Intervention - 46.8/53.2 (%) Control - 8.9/50.6 (%)	Range - <16 to >31; 32.3% smoked 16-20 cigs/day	One month

Risk of Bias Assessment

**Figure 2:**
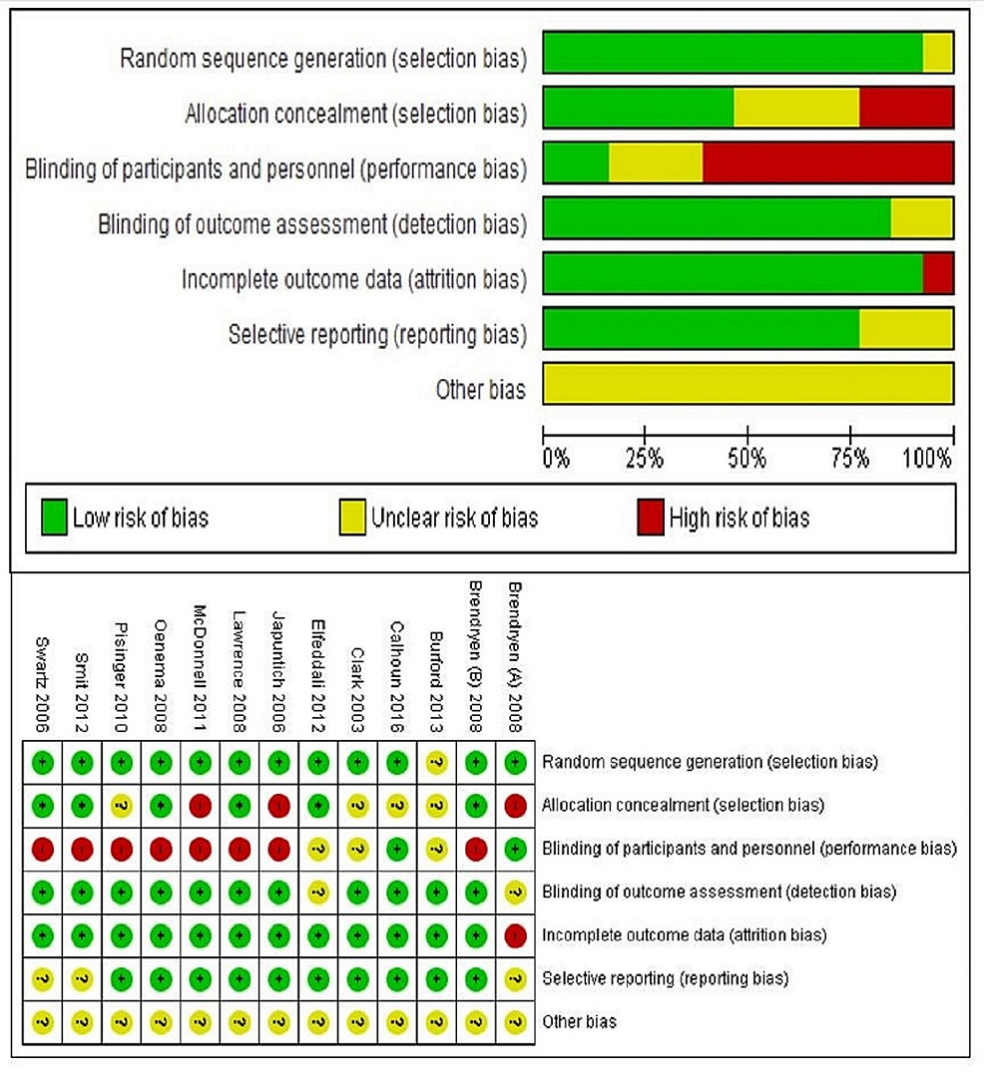
Risk of bias graph and summary The reviewers independently assessed the quality of included studies [11-23]

Two reviewers (PY and MB) independently assessed the quality of included studies. Risk of bias graph and summary has been created in Review Manager software 5.4 version under the heads of random sequence generation (selection bias), blinding of participants and personnel (performance bias), allocation concealment (selection bias), blinding of outcome assessment (detection bias), selective reporting (reporting bias), incomplete outcome data (attrition bias), and other bias [9] (Figure 2).

Data Analysis

**Figure 3:**
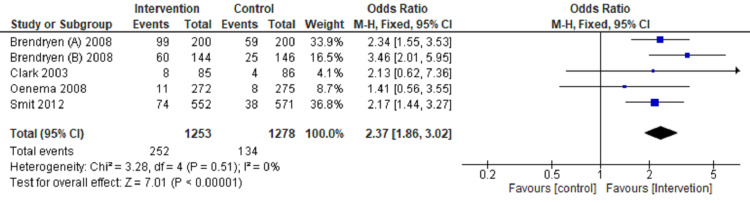
Forest plot comparing internet intervention with the control group Tobacco quit at one month follow up [11,12,14,20,22]

**Figure 4:**
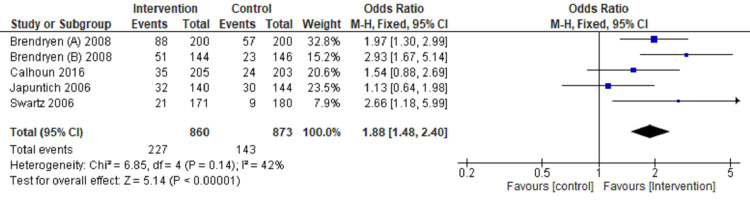
Forest plot comparing internet intervention with the control group Tobacco quit at three months follow up [11,12,15,17,23]

**Figure 5:**
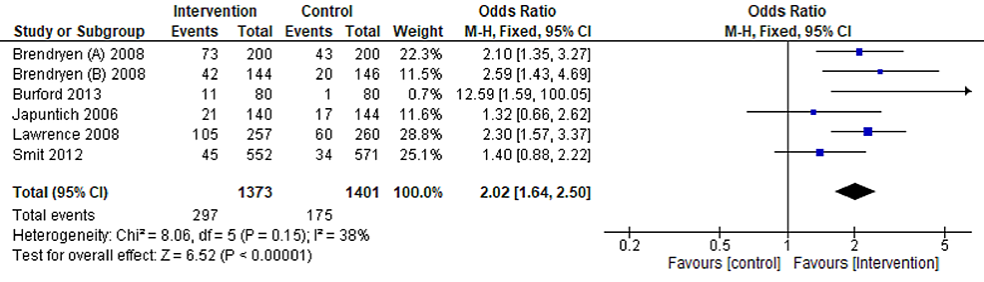
Forest plot comparing internet intervention with the control group Tobacco quit at six months follow up [11-13,17,18,22]

**Figure 6:**
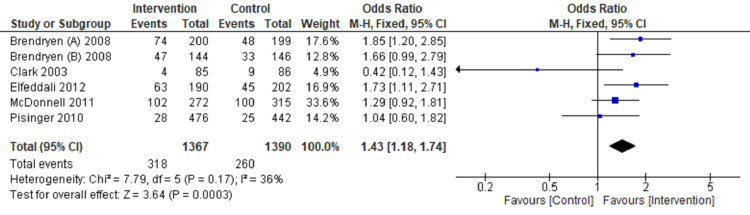
Forest plot comparing internet intervention with the control group Tobacco quit at one year follow up [11,12,14,16,19,21]

Review Manager software 5.4 version was used for meta‑analysis [9]. The fixed‑effects model and effect measures were calculated as the OR with P-value < 0.05 considered statistically significant. I2 statistics with 25%, 50%, and 75% were measured to compute statistical heterogeneity in low, moderate, and high grades-tabulated data presented in a forest plot (Figures 3-6).

**Figure 7:**
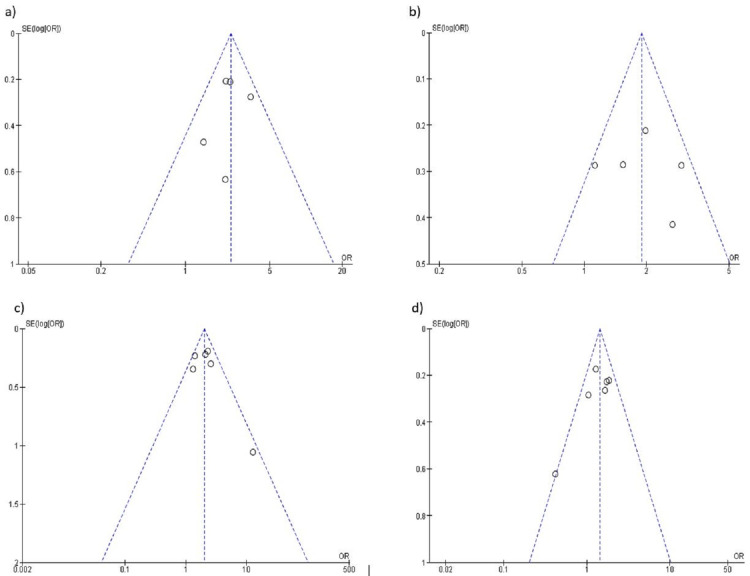
Funnel plot: shows publication bias across studies for each outcome (a) Tobacco quitting at one month, (b) tobacco quitting at three months, (c) tobacco quitting at six months, and (d) tobacco quitting at one year

The funnel plots have also been created to assess the publication bias across studies. It measures an effect estimate against its standard error for an outcome (Figure 7).

Tobacco quitting among participants has been analyzed at one, three, six, and twelve months of follow-up and presented in a forest plot. 

Results

A total of 13 articles were found suitable for meta-analysis, with 3852 and 3908 participants in intervention and control groups [11-23]. All studies revealed data with a sample size ranging from 160 [13] to 2159 [20]. Baseline characteristics of included studies have been described in Table 1. All studies have nearly equal male and female participants. Only two studies Japuntich et al. and Pisinger et al. had majority of female participants (intervention - 75.4%/control - 70.4%) and (intervention - 63.2%/control - 63.4%), respectively [17,21]. Calhoun et al. had the majority of male participants in the intervention (85%) and control group (84%) [15].

Two studies measured the outcome at four steps: one, three, six months, and one year [11,12]. Two studies followed up the participants only for one month [20,23]. Two studies measured the outcome at six months only [13,18]. Calhoun et al. measured the outcome at three months and twelve months of internet intervention and telehealth medication clinic unite with a telehealth medication clinic for nicotine replacement therapy [15]. Even four studies assessed the outcome of tobacco use at one year of different web or internet-based interventions [14,16,19,21].

Subgroup analysis with tobacco quitting outcomes at one, three, six months, and one-year follow-up further lowers the heterogeneity across studies. Sensitivity analysis was done to find a better result with a random effect model. We observed similar results with the random effect model also. Pike et al. have been removed from the analysis due to the massive difference in the number of participants in both groups, creating heterogeneity [24].

The forest plot favors the intervention group (OR: 2.37, CI: 1.86-3.02, P=0.00001, I2=0%) in comparison to the control group for quitting tobacco at a one-month follow-up (Figure 3). The forest plot also favors the intervention group compared to the control group (OR: 1.88, CI: 1.48-2.40, P=0.00001, I2=42%) for quitting tobacco at three months follow up (Figure 4). The forest plot also favors the intervention group compared to the control group (OR: 2.02, CI: 1.64-2.50, P=0.00001, I2 =38%) for quitting tobacco at six months follow up (Figure 5). The forest plot also favors the intervention group compared to the control group (OR: 1.43, CI: 1.18-1.74, P-0.00001, I2 = 36%) for quitting tobacco at a one-year follow-up (Figure 6). The forest plot suggests significantly higher tobacco quitting events in the internet intervention group at one, three, six, and twelve months of follow-up of participants with moderate heterogeneity across the studies.

Risk of bias has been assessed and created a risk of bias graph and summary of included studies under the heads of selection bias, performance bias, detection bias, attrition bias, reporting bias, and any other bias observed across the studies. It depicts that there was no serious risk of bias across the studies (Figure 2). A funnel plot has been created to estimate the effect against its standard error for included studies in each outcome (Figure 7).

Discussion

Over the year, many innovative forms of internet-based approaches, i.e., tailored computerized programs, text messages, mobile or telephone, and WhatsApp for reminder or call, app-based intervention, chat-based instant messaging, video assistance using the website and mobile and use of social media [8], have been practiced commonly to quit tobacco in different age group population. Although, various methodological issues reduce the ability to esti­mate the effects of internet-based approaches.

This study evaluated the impact of the internet approaches versus face-to-face interaction on reducing tobacco use in the adult population. Results suggest significantly higher tobacco quitting events in the internet intervention group than the control group at one month, three months, six months, and one year of follow-up of participants with moderate heterogeneity across the studies. Happy ending, a digital multi‐media smoking cessation intervention consisting of more than 400 contacts through emails, interactive voice response, Web pages, and short message service compared with self‐help booklet, reported higher point abstinence rates in the treatment group in the long-term effect of the intervention [11,12].

A written list of internet resources for smoking cessation was found more helpful than written self-help material to quit smoking for a long-term period of one year [14]. Internet-based self-help smoking cessation program, interactive, individual advice, multiple computer-tailored smoking cessation internet interventions, and a video-based internet site presented strategies for motivational materials and smoking cessation found no effect at six months of intervention but the significant effect at 12 months of follow up [19,21-23]. Personalized smoking cessation through an online life magazine in the young population enhanced smoking cessation at the end of 12 months [18].

Internet use and telehealth medication clinic combined with a telehealth medication clinic for nicotine replacement therapy reported no significant difference (17% vs. 12%) in comparison to clinical-based smoking cessation after three months of intervention [15]. However, Burford et al. compared a computer-generated photoaging intervention with no treatment group and reported a higher (27.5%) incidence of smoking quit than the control group (6.3%) at six months follow up [13]. Rabius et al. reported the follow-up response rate as 38%, and Feil et al. achieved 50% responses from participants with monetary incentives [25,26]. Findings were also reinforced by the researchers that the participants' loss inevitably influences research on the internet for health purposes [27]. After the quit attempts, web-based interventions could be more effective in preventing relapse in the long term, which requires adherence to the intervention for its effectiveness [12].

Additionally, the approach to a website supporting smoking abstinence is not related to smoking cessation [12]. Civljak et al. reported the strong effect of uniting tailored materials with nicotine replacement therapy on tobacco cessation and a significant positive impact of tailored materials among pre-contemplators [28].

The internet services should be based on their preference and easily accessible to those who want to quit smoking and seek related information through the internet, need to utilize the internet services for the same [29]. Presently, internet interventions' incremental cost is less than other modalities, facilitating and evaluating online programs' effectiveness [30]. Online interventions also can access smokers and support them in quitting tobacco, which is also firmly associated with the total and physical quality of life among adults [31].

Strength and limitations

Subgroup analysis explored and discussed the possibility of tobacco quitting in the adult population at different time points. Sensitivity analysis strengthened the evidence by exploring possible alternate findings.

Although there was a lack of uniformity of internet-based approaches in included trials, they had different internet approaches, which have also been discussed (Table 2). The risk of included bias in the individual trial also contributed towards the limitation of meta-analysis (Figure 2).

This article is available on preprint server Research Square (https://www.researchsquare.com/article/rs-318627/v1).

## Conclusions

This meta-analysis pooled the data of randomized controlled trials with a limited sample size. It winded up that internet use is highly effective in tobacco quitting at one, three, six, and twelve months of follow-up of participants compared to face-to-face intervention or no intervention with moderate heterogeneity across the studies. A moderate level of evidence supports the findings. Further studies are required to explore internet interventions' durable adherence among the adult population who spared their maximum time with the internet in any form. Additionally, limited availability of trials in developing countries requires research of internet use in developing countries to quit tobacco. Findings provide evidence to policymakers to utilize the internet as an effective instrument for tobacco control in their countries. Conclusively, this meta-analysis adds to the evidence for the promising approach of the internet-based intervention in modifying behavior, reducing tobacco use, and enhancing positive health practices among adults.
